# Nutrition rehabilitation programs and cachexia clinics for anorexia–cachexia syndrome in patients with cancer

**DOI:** 10.1093/oncolo/oyag042

**Published:** 2026-02-16

**Authors:** Rony Dev, Kunal C Kadakia, Jegy M Tennison, Koji Amano, Michele Szafranski, Eduardo Bruera, Tateaki Naito, Egidio Del Fabbro

**Affiliations:** Department of Palliative Care, Rehabilitation, and Integrative Medicine, University of Texas MD Anderson Cancer Center, Houston, TX, 77030 United States; Departments of Supportive Oncology and Medical Oncology, Levine Cancer Institute, Atrium Health, Charlotte, NC, 28262 United States; Department of Palliative Care, Rehabilitation, and Integrative Medicine, University of Texas MD Anderson Cancer Center, Houston, TX, 77030 United States; Department of Psycho-Oncology and Palliative Medicine, Osaka International Cancer Institute, Osaka University Hospital, Osaka, 541-8567 Japan; Departments of Supportive Oncology and Medical Oncology, Levine Cancer Institute, Atrium Health, Charlotte, NC, 28262 United States; Department of Palliative Care, Rehabilitation, and Integrative Medicine, University of Texas MD Anderson Cancer Center, Houston, TX, 77030 United States; Division of Thoracic Oncology, Shizuoka Cancer Center, Shizuoka, 411-8777 Japan; Department of Medicine, Medical College of Georgia, Augusta, GA, 30912 United States

**Keywords:** cancer, cachexia, anorexia, malnutrition, symptoms

## Abstract

The anorexia–cachexia syndrome (ACS) is characterized by loss of appetite and unintentional weight loss. Important clinical outcomes are associated with ACS including increased risk of chemotherapy side effects, decreased survival, and quality of life. Because ACS is driven by complex metabolic mechanisms and a chronic pro-inflammatory response, the weight loss and muscle wasting cannot be reversed by conventional nutritional supplementation alone. However, insufficient intake of calories and protein exacerbate weight loss experienced by patients with ACS, while physical inactivity accelerate muscle wasting. In addition, uncontrolled symptoms, such as pain, mucositis, nausea, early satiety, and depression aggravate poor nutritional intake and are known as nutrition impact symptoms. Addressing these potentially reversible contributors to ACS requires an interdisciplinary team (IDT) effort including oncologists, palliative medicine, rehabilitation clinicians, dietitians, and psychologists. The composition and leadership of the team depends on institutional support and the patient population being treated (eg, advanced cancer vs peri-operative rehabilitation vs geriatric). Because patients may be burdened by frequent visits to multiple healthcare providers and a special skill set is required of the IDT to address ACS—measuring caloric and protein intake, assessing body composition, optimal symptom management, and providing psycho-social support–a specialized clinic would be ideal. As more effective anti-cachexia agents are being developed, nutritional rehabilitation programs and cachexia clinics could facilitate incorporation of novel treatments into multimodal management of ACS. The narrative review highlights the management of nutrition impact symptoms and the potential benefits and challenges of specialized nutrition rehabilitation programs and cachexia clinics.

Implications for Practice Anorexia–cachexia syndrome (ACS) in patients with cancer is associated with decreased quality of life, increased mortality and cannot be fully reversed by conventional nutritional support. Effective treatment for patients with ACS requires screening for malnutrition, evaluation of body composition and functional status, and a thorough assessment and treatment of symptoms that impact caloric intake. Treatment of a single domain, such as insufficient caloric intake or anorexia alone, may alleviate an individual symptom but may not be adequate to maintain or reverse weight loss or reduce distress in patients with ACS. Clinical researchers advocate for multimodal interventions by an interdisciplinary team in specialized nutrition rehabilitation programs and cachexia clinics.

Key recommendations for the treatment of anorexia–cachexia syndrome in patients with cancer.

- Anorexia–cachexia syndrome (ACS) is characterized by loss of appetite and unintentional weight loss.
- Muscle wasting and a decline in muscle quality and performance are features of ACS.
- ACS is associated with functional decline, poor survival, and decreased QOL.
- Signs of wasting and loss of appetite can cause psychosocial distress in both patients and family caregivers.
- Ideal assessments for ACS include malnutrition screening, assessment of protein and caloric intake, monitoring weight, physical performance, and if possible, body composition.
- Simple screening criteria for anorexia include the Edmonton Symptom Assessment System appetite score of ≥3/10 and for cancer cachexia include >5% weight loss from baseline.
- Uncontrolled symptoms such as nausea, mood disorders, early satiety, or pain may decrease caloric intake and managing these nutrition impact symptoms may improve oral intake and mitigate weight loss.
- Inexpensive treatment such as metoclopramide for early satiety, laxatives to prevent constipation, and olanzapine for anorexia may mitigate weight loss.
- ACS is difficult to treat and ideally needs an interdisciplinary team approach including a dietitian, physical therapist, psychologist, oncologist, and palliative care professionals.
- Behavioral counseling and psychological support may promote treatment adherence and adaptive coping to stress.
- Preliminary studies combining exercise and nutritional counseling with multimodal interventions targeting the underlying mechanism of ACS show promise.
- Nutrition rehabilitation and cachexia clinics should be considered in cancer centers with adequate resources to optimize treatment of ACS.

## Introduction

The anorexia–cachexia syndrome (ACS) is characterized by loss of appetite and unintentional weight loss and is associated with increased risk of chemotherapy side-effects, poor survival, diminished function, and decreased quality of life (QOL).^1^ Because ACS is driven by complex metabolic alterations and a chronic pro-inflammatory response, the weight loss and muscle wasting cannot be reversed by conventional nutritional supplementation alone. However, insufficient intake of calories and protein may exacerbate the weight loss experienced by patients with ACS.

Improving nutrition intake is possible by addressing symptoms that contribute to anorexia and muscle wasting. Symptoms commonly encountered in patients with cancer, such as nausea, depressed mood, early satiety, or pain, may decrease oral intake and are referred to as nutrition impact symptoms (NIS). The ASCO guidelines for cancer cachexia support the concept of addressing NIS, stating “uncontrolled symptoms of cancer or its treatments” “can be detrimental to food intake.”^1^ By improving nutritional intake and promoting physical activity, the muscle wasting, and adverse outcomes associated with ACS may be mitigated.

Although managing NIS is a key component, other factors such as physical inactivity, eating-related conflict between patients and family members or inadequate access to high-quality nutrition may play an important role in exacerbating ACS. Additional non-symptom barriers can also impede dietary intake (even after nutritional counselling), such as conflicting advice and personal food restrictions.^2^ Managing these potentially reversible clinical and psychosocial domains requires a collaborative, interdisciplinary team (IDT) involving physicians, nurses, dietitians, physical therapists, and clinical psychologists. This multimodal approach to ACS is especially important given current ASCO guidelines are unable to recommend a specific pharmacological intervention for cachexia as standard of care.

The primary aim of this narrative review is to provide an overview of pragmatic strategies for managing ACS, such as exercise and nutrition, malnutrition screening, and the assessment of NIS and other contributors and treatment opportunities. In addition, research on nutrition rehabilitation programs and cachexia clinics will be summarized.

## Assessments

In patients with cancer, the ideal assessments for ACS include malnutrition screening and measuring protein and caloric intake, monitoring weight, body composition and performance status (PS), and assessing NIS.^3^ The feasibility of completing all these assessments depends on institutional support and team resources.

### Malnutrition screening

Several validated^4^ screening tools are available for malnutrition in patients with cancer, including the Patient-Generated Subjective Global Assessment Short Form (PG-SGA SF),^5^ Malnutrition Screening Tool,^6^ and the Malnutrition Universal Screening Tool (MUST).^7^ There is no consensus on the best tool for screening, since each tool has merit but also some limitations.^8^ The PG-SGA SF for example, has predictive value for survival, and can be used as a screening, assessment or monitoring instrument. However, the PG-SGA-SF does not include an assessment of body mass index (BMI), a simple, yet important measure that should be done at each visit. The initial BMI and the rate of weight loss predict survival in patients with solid tumors independently of conventional factors such as cancer site, stage, and PS.^9^ Additional measures of anorexia severity may be useful to measure longitudinally—these include an appetite score of ≥3/10 on the Edmonton Symptom Assessment System (ESAS) or ≤37 on the Anorexia/Cachexia Subscale of the Functional Assessment of Anorexia/Cachexia Therapy (FAACT-A/CS).^4^

### Nutrition impact symptoms

Studies of patients across various tumor types and treatments show individual NIS and/or their aggregate number are associated with adverse clinical outcomes. While the treatment of an individual NIS may not reverse weight loss or modulate the mechanisms driving ACS, managing NIS may slow wasting and improve QOL.

More severe anorexia is associated with greater weight loss, poorer performance status and higher prevalence of NIS such as early satiety, constipation, and vomiting.^10^ A systematic review of 30 anti-neoplastic treatment trials found more severe anorexia or pain scores at baseline were associated with decreased survival.^11^ A multi-center study from Japan reported taste and smell disturbances were associated with worse dietary intake and QOL scores, independent of performance status and degree of ACS.^12^ A cross-sectional study of outpatients at high risk for malnutrition showed the combination of anorexia and early satiety compared to anorexia alone was associated with significantly worse weight loss, worse overall health perception, and fatigue.^13^ In surgical patients with cancer, the number of NIS is associated with higher prevalence of malnutrition,^14^ and a longitudinal study of patients with esophageal cancer found more NIS were associated with worse QOL and physical function at 6 months after surgery, regardless of the pre-operative BMI or post-operative weight loss.^15^ Similarly, aggregate burden of symptoms prior to radiation and/or chemotherapy was a significant independent predictor of reduced intake, weight loss, and survival in patients with head and neck cancer.^16^

Constipation is common in patients receiving opioids or ondansetron and underappreciated as a NIS. In patients with early-stage head and neck cancer receiving radiation therapy, constipation was associated with significantly greater weight loss,^17^ while a prospective study of patients with newly diagnosed esophageal cancer found symptoms of anorexia and constipation at the start of chemoradiation persisted 4-6 weeks after completion of therapy. Long-term survivors are also at risk of adverse effects that correlate with NIS burden. In a population-based prospective study found symptoms of pain, fatigue, nausea and vomiting, and appetite loss were associated with a > 15% weight loss 5 years after surgery for esophageal cancer.^18^

#### Assessment

Brief (<5 minutes) questionnaires to assess NIS include the PG-SGA SF and the ESAS. ESAS has the advantage of providing a numeric rating scale (0-10) for individual symptoms but evaluates fewer ACS symptoms than the PG-SGA SF. A questionnaire derived from both ESAS and PG-SGA evaluated NIS and eating-related distress in Japanese patients with advanced cancer and their families. Patients with ACS had significantly increased eating-related distress, tiredness, drowsiness, lack of appetite, early satiety, diarrhea, abnormal taste, and difficulty swallowing compared to patients without ACS.^19^^,^^20^

#### Management

Few studies characterize the medical management of individual NIS in patients with cancer. Some medications may be effective for multiple symptoms, for example, duloxetine (neuropathic pain + depression), mirtazapine (depression + non-CINV nausea), and olanzapine (CINV and non-CINV nausea + resistant depression).^21^ Observational studies indicate that prokinetic metoclopramide may be useful for early satiety/gastroparesis and non-CINV. An early study using the PG-SGA identified multiple unmanaged symptoms in patients with cancer, including loss of appetite, nausea, early satiety, dry mouth, and altered taste, which resulted in the incorporation of dietary counseling, prescriptions for artificial saliva, and the addition of metoclopramide.^22^ Laxatives and metoclopramide were the most common medications used in 151 patients with advanced cancer seen by the MD Anderson Cancer Cachexia Clinic.^23^ Notably, conventional appetite stimulants such as corticosteroids or megestrol acetate were not prescribed, yet one third of patients experienced weight gain.

### Body composition

Body composition (lean mass and fat mass) assessments may be useful for diagnosis, prognosis and for monitoring the efficacy of interventions. Decreased muscle mass (sarcopenia) is associated with a poor prognosis in patients with solid tumors and highly prevalent in patients admitted to the ICU with hematological malignancies.^24^ The combination of reduced lean body mass and excess adiposity (sarcopenic obesity) is associated with a particularly poor prognosis in patients with solid tumors and is difficult to identify without imaging.^25^ Computed tomography scans offer an accurate, opportunistic evaluation of body composition at diagnosis or re-staging. However, dual-energy-x-ray absorptiometry (DXA) is still considered the gold standard, although lower-cost evaluations with bio-electrical impedance analysis (BIA) and anthropometrics may be more practical for routine clinical use. DXA is most accurate, but multi-frequency BIA is a convenient, relatively lower-cost body composition assessment tool that can be used longitudinally to measure fat-free mass and fat mass.^26^

### Laboratory tests

A limited set of labs should be considered based on clinical history and possible co-morbid conditions contributing to wasting including C-reactive protein, albumin, vitamin B12 and D, thyroid function, and testosterone.

### Physical performance

Muscle wasting and a decline in muscle quality and performance are features of most patients with ACS. A systematic review^27^ of functional assessments in cachexia trials included 5 objective outcomes: Hand grip strength (HGS), stair climb power, timed up and go test, 6-minute walking test, Short Physical Performance Battery (SPPB) and chair stand test (time needed to rise 5 times from a seated position without a patient using their arms). HGS (with a dynamometer) was the most used physical function endpoint; however, the authors could not comment on which tool was optimal. In older patients with cancer, higher symptom burden is associated with functional impairment. For example, in 359 patients (median age 81 years), each unit increase in a composite symptom score was associated with greater activity of daily living impairment, physical activity limitations, falls, and SPPB ≤9 (*P* < .05).^28^ The SPPB is predictive of clinical outcomes, including mortality and health care utilization and includes a composite test of gait speed, balance, and a chair stand test (approximately 10 minutes to complete). A SPPB ≤9 score is predictive of decreased overall survival in older patients with leukemia,^29^ increased mortality in older women with gynecological cancer,^30^ and lower chemotherapy completion in non-small cell lung cancer.^31^

### Patient and caregiver distress

Physical signs of wasting and loss of appetite can lead to conflict among patient and family over caloric and food intake, body image dissatisfaction, and existential distress or anticipatory grief.^32^ Family caregivers’ eating-related distress may be greater than patient eating-related distress.^33^ A qualitative study involving 31 patient-spouse/partner dyads using semi-structured interviews found interactions between patient and family caregivers can either sustain or ameliorate eating-related distress.^34^ A recent scoping review found 3 ways a psychosocial component of multimodal management could help patients with ACS. These include behavioral counseling to promote treatment adherence, adaptive coping to emotional stress, and managing anxiety and depression.^35^

### Key components of nutrition rehabilitation programs and cachexia clinics

Nutrition rehabilitation programs and cachexia clinics should ideally incorporate key components of nutritional counseling, exercise, pharmacological interventions, and psychosocial support. The team composition may vary depending on resources; however, a dietitian is usually a core member of the IDT. The inclusion of physical therapists and psychologists with expertise in oncology is desirable.^36^ A single visit with a multidisciplinary team would be preferable, so as not to place an undue burden on patients. If interdisciplinary members are not “embedded” within the clinic, patients could be seen in tandem.

The composition and leadership of the IDT will depend on institutional resources and strengths, coupled with patient needs. For example, patients with high symptom burden and advanced disease would benefit from a palliative medicine specialist with expertise in symptom management and opioid use, while an older patient may best be served by a team with expertise in aging (eg, Geriatric oncology or Endocrinology). Nutritional rehabilitation programs may serve a broader patient population than cachexia clinics, by including patients preparing for surgery with “prehabilitation” programs and long-term survivors of serious illness who may have completed disease directed therapy. Integration of clinics within an already established supportive/palliative care centers, rehabilitation medicine clinics, subspecialty oncology program, or a collaborative effort within specific cancer types at greater risk for cachexia can potentially provide more rapid access, streamline referrals, and identify motivated patients who can adhere to multi-modality treatment interventions.

### Challenges

Despite promising data and an intuitive appreciation of nutrition and exercise health benefits, challenges remain in establishing and sustaining cachexia clinics and nutrition rehabilitation programs. Early inclusion of dietitians, physiotherapists and psychologists during the planning and pilot phase should be considered to reinforce shared vision and goals for the specialty clinic and provide consistency of information and treatment recommendations. Educating colleagues about the impact of ACS on functional outcomes and adopting screening tools for malnutrition and ACS to facilitate referral to specialty clinics is necessary. An international survey of healthcare professionals noted only 29.1% recognized >5% weight loss from baseline as a key criterion for cancer cachexia, and only 47.4% reported weighing patients at each visit.^37^ Care pathways adopting simple referral criteria that include weight loss (eg, ≥ 5% within past 6 months or ≥2.5% in patients with BMI < 20) or symptoms (ESAS appetite score ≥3) or PG-SGA SF scores ≤37 may increase awareness among clinicians and encourage referrals. A Japanese survey found healthcare professional did not regularly provide education and emotional support to either patients suffering from cancer cachexia or their families.^38^ These studies highlight the need for more education regarding assessment and treatment of ACS and the need for specialty clinics. However, the burden of clinic evaluations, such as patient-reported outcomes, body composition, diet intake diaries, and functional assessments, should not overwhelm patients.

## Nutrition rehabilitation and cachexia clinics in cancer centers

Cachexia clinics and combined exercise and nutrition rehabilitation programs have been established within outpatient oncology centers for over a decade. The goals of these programs are to focus on the complex needs of patients with cancer who have ACS with a systematic multimodal approach utilizing an IDT. Table 1 provides an overview of 12 published studies combining nutrition + exercise along with medications (either targeting NIS or selected mechanisms of ACS). Some of the studies are from nutritional rehabilitation programs or cachexia clinics. Their design, number of participants, methods, outcome measured, and main findings are outlined. Retrospective (*n* = 4) and non-randomized prospective (*n* = 6) studies evaluating the impact of these specialty clinics suggest variable benefits in improved symptom control,^23^^,^^39–44^ increased functional capacity,^39–41^^,^^42^^,^^44^^,^^45^ enhanced nutritional status,^23^^,^^39–44^ and less psychosocial distress.^39^^,^^41^^,^^42^^,^^45^

**Table 1.** Overview of published studies on nutrition rehabilitation programs and cachexia clinics in patients with cancer.

Author and year/intervention/aim(s)	Participants/setting	Outcome measures (time points)	Main findings and effects	Strengths/limitations
**Randomized controlled trials**				
**Uster et al. 2018** ^46^ **12-week nutrition and physical exercise program vs usual care** **Primary endpoint was global health status/quality of life measured using the EORTC QLQ-C30 at 12 weeks. Key secondary endpoints included difference in dietary intake, physical performance, and nutritional status.**	*N* = 58, Stage III-IV GI or thoracic cancers Outpatient clinic (Zurich, Switzerland)	Symptoms: EORTC QLQ-C30 Functional: HGS, 30-second chair sit-to-stand test, 6MWT, 1 repetition maximum leg press, ECOG PS Nutrition: 3-day food diary, NRS-2002, bioelectrical impedance analysis Psychosocial: Nil Labs: Nil (Pre and at 3 and 6 months)	Feasibility: Median adherence to the supervised exercise program was 75%, however, poor accrual led to early closure. Symptoms: No difference in global health status/quality of life was observed (change in EORTC QLQ-C30 was 4.5 ± 3.4 [intervention] vs 2.7 ± 4.0 [control], p = 0.72). Intervention arm had greater improvement in nausea and vomiting subscale. Functional: No statistical differences observed for any measure of physical performance. Nutrition: Intervention arm had greater protein intake but no difference in overall nutritional status by BIA or weight	Strengths: Randomized trial of a combined nutrition and exercise program with multiple functional and nutritional endpoints. Confirmed feasibility of trial components in study population. Though no statistically significant differences, intervention group had numerical improvements in nearly all endpoints. Limitations: Poor accrual led to early closure and was under powered for primary endpoint (447 screened, 161 met inclusion, and 101 refused to participate).
**Lu et al. 2021** ^47^ **Early interdisciplinary supportive care (ESC) vs usual care** **To compare the clinical benefits of early interdisciplinary supportive care (ESC), which included nutritional and psychologic support, with usual care. Primary endpoint was overall survival and second endpoints including change in quality of life, objective response rates, and adverse events.**	*N* = 328, Stage IV gastroesophageal cancer Outpatient clinic (Beijing, China)	Symptoms: EORTC QLQ-C30, NCI-CTCAE 4.0 Functional: HGS, 30-second chair sit-to-stand test, 6MWT, 1 repetition maximum leg press, ECOG PS Nutrition: NRS-2002, PG-SGA Psychosocial: DT, HADS-A, HADS-D, PHQ-9 Labs: Nil (pre and throughout study period)	Primary endpoint: Median overall survival was improved with ESC than usual care, 14.8 (95% CI 13.3-16.3) vs 11.9 (9.6-13.6) months, *P* = .021. Overall survival improvement observed in preplanned exploratory analysis according to the primary tumor site. Progression-free survival, objective response rate, and adverse events were not significantly different between the groups. Symptoms: From baseline to week 9, ESC group had a significant improvement in emotional and cognitive functioning. Nutrition: From baseline to week 9, ESC group had a significant improvement in NRS 2002 and PG-SGA scores. Less patients had weight loss in ESC grout (45vs 58%, *P* = .032) Psychosocial: From baseline to week 9, ESC group had a significant improvement in DT, HADS-A, HADS-D, and PHQ-9.	Strengths: Primary endpoint was overall survival. Large randomized controlled trial in homogenous advanced cancer population. A simple ESC model for further study Limitations: Limited external validity as conducted at single intuition in China. Not blinded. Moderate drop-out in both nutrition (33%) and psychological (28%) interventions over 9-week study period.
**Non-randomized prospective studies**				
**Granda-Cameron et al. 2010** ^58^ **12-week Cancer Appetite and Rehabilitation (CARE) Clinic** **Evaluate the nutritional and symptom distress for patients who attended the CARE Clinic at least 4 times (*N* = 11) and assess patient satisfaction (*N* = 25).**	*N* = 96, any stage, heterogenous cancers Outpatient clinic (Pennsylvania, USA)	Symptoms: ESAS Functional: KPS Nutrition: Weight, body cell mass, caloric goal Psychosocial: Nil Labs: CBC, renal/liver function, albumin, pre-albumin, testosterone level, and Vitamin D (Pre and Post)	Feasibility: “Overall, most patients felt that the CARE Clinic was a worthwhile experience and all patients said they would recommend the clinic to a friend” Nutrition and Symptoms (no data provided): “Although not statistically significant, a trend for improvement was observed between visit 1 and visit 4 (12 weeks) in weight, body cell mass, and appetite levels”	Strengths: Comprehensive description of the interdisciplinary assessment and treatment goals conducted by the medical, nursing, nutrition, physical therapy, and speech and swallowing teams. Limitations: Small sample size of the evaluable population (*N* = 11). Limited results provided within publication. No control group.
**Chasen et al. 2010** ^39^ **8-week Cancer Nutrition and Rehabilitation Program (CNRP)** **Evaluate if CNRP has an effect on symptoms and quality of life**	*N* = 53, Stage II-IV gastroesophageal cancer Outpatient clinic, (Montreal, Canada)	Symptoms: ESAS, BFI Functional: 6MWT Nutrition: PG-SGA Psychosocial: DT Lab: Nil (Pre and Post)	Symptoms: Improvements in ESAS of appetite, strength, nervousness, depression, pain, constipation, and nausea (all *P* = <0.05). Fatigue by BFI improved in enjoyment of life, general activity, usual fatigue, and fatigue now. Functional: Median 6MWT (range) improved from 384 m (173-570) to 435 m (203-630), *P* = .01 Nutrition: Median PG-SGA (range) improved from 12 (2-24) to 9 (1-18) Psychosocial: Median DT (range) improved from 4 (1-7) to 2 (0-6), *P* = .01	Strengths: Single cancer type. Use of validated tools. Limitations: High dropout rate (58%) due to disease progression and inability to attend regularly. No control group.
**Glare et al. 2011** ^57^ **8-week Cancer Nutrition and Rehabilitation Program (CNRP)** **To demonstrate feasibility and satisfaction of a CNRP and to determine benefits and outcomes**	*N* = 54, heterogenous cancers (stage not provided) Outpatient clinic (Sydney, Australia)	Symptoms: ESAS Functional: KPS, 6MWT, HGS Nutrition: PG-SGA, Body composition analysis Psychosocial: Nil Labs: Albumin, CRP (Pre and at 1, 2, 3, and 6 months)	Feasibility: 68%, 58%, 44%, and 12% compliance at 1, 2, 3, and 6 months. >90% patient reported CNRP as important. Likelihood for returning at the 2-month follow-up were higher 6MWT, lower ESAS score, active anticancer therapy, better KPS, less weight loss, male gender, low CRP, low GPS, low PG-SGA, and higher albumin	Strengths: Provided predictors of compliance to a CNRP program at a high-volume cancer center Limitations: No formal statistical testing on effect of CNRP on outcomes. Despite collection of body composition data, no data provided of CNRP effect on body composition. No control group.
**Chasen et al. 2013** ^40^ **8-week Palliative Rehabilitation Program (PRP)** **To evaluate predictors of program completion and changes in functioning, symptoms, and well-being.**	*N* = 116, Stage III-IV heterogenous cancers (completed anticancer therapy) Outpatient clinic (Ottawa, Canada)	Symptoms: ESAS, MFI Functional: ECOG PS, MDASI-Impact on Function Subscale, Berg Balance Scale, Functional Reach Test, Timed Up and Go, HGS, 6MWT Nutrition: PG-SGA Psychosocial: Nil Labs: CBC, electrolytes, CRP, albumin, TSH, glucose, and LDH (pre and post)	Feasibility: 58% completed the 8-week program. Reasons for non-completion included disease progression, personal, death, or too well. Likelihood to completing program was higher if baseline CRP <10. Symptoms: Moderate-to-large effects were observed in anxiety, depression, overall well-being, feeling tired, and fatigue (effect size = 0.38-0.55) Functional: Improvement in 6MWT (effect size = 0.80), Time up and Go (effect size =0.65), and Functional Reach Test (effect size = 0.44). No statistical difference in Berg Balance scale or HGS. ECOG PS improved (effect size = 0.9) Nutrition: Significant improvement in overall nutritional risk (effect size = 0.46).	Strengths: Exhaustive use of validated functional assessments. Limitations: No results on weight change. No control group.
**Gagnon et al. 2013** ^41^ **10-12-week Cancer Nutrition-Rehabilitation (CNR)** **To evaluate predictors of program completion and changes in functioning, symptoms, and well-being.**	*N* = 188, Stage III-IV heterogenous cancers Outpatient clinic (Montreal, Canada)	Symptoms: ESAS, MFI Functional: 6MWT and 5-m Walk Test, ECOG PS Nutrition: Weight history Psychosocial: DT and CT Labs: CRP, albumin (pre and post)	Feasibility: 70% completed the 10-12-week CNR. Reasons for non-completion included disease progression, dropped out, and death. Likelihood of non-completion was higher if baseline ECOG PS was poor, elevated CRP >20, poor nutritional status, and worse anorexia. Symptoms: Moderate reduction in weakness, depression, nervousness, and shortness of breath as well as reduced activity, physical and general fatigue (effect sizes = 0.5-1.1). Functional: Mean 6MWT at baseline was 395 ± 111 m, increased by 41 m (95% CI, 29-52, effect size, 0.7). Maximal gait speed at baseline was 1.5 ± 0.44 m/s, increased by 0.15 m/s (95% CI, 0.09-0.21, effect size, 0.6). Nutrition: 77% gained or maintained weight. Psychosocial: Moderate reduction in distress and coping (effect sizes = 0.5-0.7)	Strengths: Large multi-year experience with high program completion rates. Detailed description of interventions provided by each cancer nutrition-rehabilitation health professional (Physician, RN, Dietician, OT) Limitations: No long-term results. No control group.
**Feldstain et al. 2016** ^45^ **8-week Palliative Rehabilitation Program (PRP)** **To examine the impact of 3 PRP factors (inflammation, exercise, and self-efficacy) on depression in those completing the program (*N* = 80)**	*N* = 131, Stage III-IV heterogenous cancers Outpatient clinic (Ottawa, Canada)	Symptoms: Nil Functional: 6MWT Nutrition: Nil Psychosocial: GSE, HADS Labs: CRP (pre and post)	Functional: Mean 6MWT at baseline was 372.55 m (*SD* 137.71), increased to 412 m (*SD* = 144.31), *P* = <.001 Psychosocial: Self-efficacy (GSE) significantly increased from 27.86 (*SD* = 6.16) to 31.23 (*SD* = 5.77) and depression decreased from 7.14 (*SD* = 3.91) to 5.95 (*SD* = 3.51), *P* = .002. Lab: Inflammation, as measured by CRP, did not significantly change.	Strengths: Analyzed pre-specified factors felt to be contribute to depression and observed self-efficacy as largest contributor. Limitations: No nutritional data. No control group.
**Retrospective studies**				
**Del Fabbro et al. 2011** ^23^ **Dietary counseling and exercise recommendations within an interdisciplinary cachexia clinic** **To determine the frequency and type of contributors to weight loss and anorexia, their respective intervention, and the effect of the cachexia clinic on clinical outcomes**	*N* = 151, Advanced heterogenous cancers Outpatient clinic (Texas, USA)	Symptoms: ESAS and S-NIS Functional: Nil Nutrition: PG-SGA and bedside indirect calorimetry Psychosocial: Nil Labs: Vitamin B12, TSH, total testosterone, and serum cortisol at baseline. (Pre and at follow-up)	Symptoms: Median S-NIS was 3 and 15% experienced ≥5 S-NIS (most commonly, early satiety, constipation, N/V, and mood changes). Most common interventions were metoclopramide, laxatives, antidepressants, and zinc. Nutrition: Significant improvements of appetite between visits with 34% gaining weight. 42% were hypermetabolic as measured by indirect calorimetry. Labs: Adrenal insufficiency, hypothyroidism, and B12 deficiency were infrequently observed, however, hypogonadism was present in 73% of men.	Strengths: Detailed description of secondary nutritional impact symptoms and corresponding intervention Limitations: Moderate drop out (39%) due to death or hospice, severe symptoms, noncompliance, or residence out of state. No functional evaluation. No control group
**Eades et al. 2013** ^42^ **8-week Cancer Nutrition-Rehabilitation Program (CNR)** **To evaluate the extent CRN affects nutritional and functional status**	*N* = 27, Stage I-IV H/N cancer after completion of combined modality treatment Outpatient clinic (Montreal, Canada)	Symptoms: ESAS Functional: MDASI-Impact on Function Subscale, 6MWT Nutrition: Body weight and presence of PEG feeding tube Psychosocial: DT Lab: Nil (pre and post)	Symptoms: Improvement in pain, quality of life, weakness, shortness of breath, anorexia, insomnia, depression (effect sizes = 0.6-0.9) Functional: No significant effect on MDASI-Function Subscale. Mean 6MWT at baseline was 421 ± 99 m, increased by 59 m (95% CI, 27-91, effect size, 0.8). Nutrition: 78% either gained or maintained weight. Of 11 patients who needed PEG feeding, 8 continued at end of program. Psychosocial: Mean change in DT (95% CI) improved 1.6 (0.7-2.5), effect size = 0.7.	Strengths: Single cancer type. Detailed description of interventions provided by each cancer nutrition-rehabilitation health professional (physician, RN, dietician, OT) Limitations: Minimal objective assessment of nutritional status. Although taste/smell alterations and xerostomia were collected at baseline, not collected at follow-up. No control group.
**Parmar et al. 2017** ^43^ **McGill Cancer Nutrition Rehabilitation Program clinic at the Jewish General Hospital (CNR-JGH)** **To measure the impact on quality of life of a multimodal rehabilitation program for cancer cachexia attending the CNR-JGH**	*N* = 374, Stage III-IV heterogenous cancers Outpatient clinic (Montreal, Canada)	Symptoms: FAACT Functional: 6MWT, ECOG PS Nutrition: Weight history Psychosocial: Nil Labs: CRP, albumin (pre and post)	Feasibility: 42% of the original cohort remained at the third visit. Likelihood for completion of 3 visits was non-lung cancer type, lower baseline CRP, and better baseline quality of life scores. Symptoms: Statistically significant improvements in FAACT total and specific subscales (physical well-being, anorexia-cachexia. No difference in emotional or social well-being subscales. Functional: Patients who increased 6MWT had greatest improvements in quality of life. Nutrition: Patients who gained weight had greatest improvements in quality of life. Labs: Baseline CRP was not associated with quality of life.	Strengths: Large cohort to identify characteristics associated with quality-of-life improvement during a multimodal rehabilitation program for cancer cachexia. Limitations: No specific details on change in functional and nutritional provided (only correlations with quality-of-life survey was provider). No control group
**Vaughan et al. 2020** ^44^ **Barwon Health Cachexia and Nutrition Support Service (CNSS)** **To characterize the CNSS service model and describe patient attendance and available nutrition and functional changes for those with ≥2 visits**	*N* = 175, Stage I-IV heterogenous cancers Outpatient clinic (Victoria, Australia)	Symptoms: Nil Functional: HGS, 30-second chair sit-to-stand test Nutrition: Weights Psychosocial: Nil Labs: CRP, albumin, hemoglobin, testosterone	Feasibility: 236 patients referred, 61 excluded from analysis primarily due to insufficient data and nonattendance. Of the 175 patients, 46% referred by oncologist, 39% by local medical office, and 11% from palliative care professional with median time-to-referral from initial cancer diagnosis of 12.9 months (0.4-304 months). 42% did not return for follow-up with most common reasons being death. Functional: 4% had increase in HGS and 38% had increase in Sit-to-Stand score. Nutrition: 78% had stabilization and 31% increased weight. Labs: 29% had increase in albumin.	Strengths: Detailed description of pharmacologic interventions provided by palliative care physician. Limitations: Nutritional and functional results are biased by significant attrition of participants. Labs changes beyond albumin not provided. No control group

Abbreviations: 6MWT, 6 minute walk test; BFI, Brief Fatigue Inventory; CBC, complete blood count; CRP, C-reactive protein; CT, Coping Thermometer; DT, Distress Thermometer; ECOG PS, Eastern Cooperative Oncology Group Performance Status; EORTC QLQ-C30, European Organization for Research and Treatment of Cancer Quality of Life Questionnaire version 3.0; ESAS, Edmonton Symptom Assessment Scale; FAACT, Functional Assessment of Anorexia-Cachexia Therapy; GPS, Glasgow Prognostic Score; GSE. General Self-efficacy Scale; HADS, Hospital Anxiety and Depression Scale; HGS, hand-grip strength; KPS, Karnofsky Performance Status; LDH, lactate dehydrogenase; m, meters; MDASI, MD Anderson Symptom Inventory; MFI, Multidimensional Fatigue Inventory; NCI-CTCAE 4.0, National Cancer Institute Common Terminology Criteria for Adverse Events version 4.0; NRS-2002, Nutrition Risk Screening 2002; OT, occupational therapist; PEG, percutaneous endoscopic gastrostomy; PG-SGA, Patient-Generated Subjective Global Assessment; PHQ-9, Patient Health Questionnaire-9; S-NIS, Secondary Nutrition Impact Symptoms; TSH, thyroid stimulating hormone.

### Evidence for combined interventions

While prospective randomized controlled trials (RCTs) on the impact of cachexia clinics and nutrition rehabilitation programs are limited, a few trials have tested the effects of a combined nutrition and physical exercise interventions.^46^^,^^47^ A systematic review examined the effect of exercise and nutritional interventions on body composition in patients with advanced or metastatic cancer (*n *= 8). The authors concluded that both intervention approaches preserve lean mass, while only combined interventions may lead to alterations in fat mass.^48^ No RCTs include NIS in their multimodal treatment; however, 2 components of the multimodal approach (dietitian and psychologist) were used to compare early interdisciplinary supportive care (ESC) vs usual care in 328 patients with metastatic esophagogastric cancer.^47^ Nutritional risk screening included the PG-SGA and interventions were daily targets of 20 kcal-30kcal/kg, daily protein intake at 1 g-1.5 g/kg of body weight, and enteral or parenteral nutrition as indicated. Psychologists conducted depression and anxiety screening and provided individual and family psychotherapy. ESC vs usual care showed improved overall survival and better PG-SGA and depression scores, and fewer patients had weight loss.

Additional studies are worth noting. A RCT determining the effect of multimodal prehabilitation vs postoperative rehabilitation for Frail Patients Undergoing Resection of Colorectal Cancer found no benefit in the Prehabilitation group despite a personalized, supervised, and home-based multimodal program prescribed by a kinesiologist, a nutritionist, and a psychology-trained nurse. The authors speculate that the limited duration of the intervention (4-5 weeks) may be one reason for the result.^49^ Another preliminary study of early prehabilitation in patients with lung cancer demonstrated feasibility and incorporates a palliative medicine physician plus registered dietitian and rehabilitation physiotherapist into the IDT.^50^

Two preliminary trials in patients with advanced cancer, from Japan (single arm)^51^ and the United Kingdom (randomized)^52^ using a combination of exercise and nutritional support reported feasibility and improved outcomes. A guideline-directed, physician-led Cancer Nutrition Program at a Comprehensive cancer center in France, improved clinical outcomes and decreased costs in patients with advanced cancer. Over a 3-year period, the investment in developing a team that included cancer-specific training of dieticians, decreased the number of patients receiving parenteral nutrition from 157 to 67, significantly increased the enteral/parenteral patient ratio, and reduced costs for nutritional care by 54%.^53^

### Future directions

**Figure 1.**
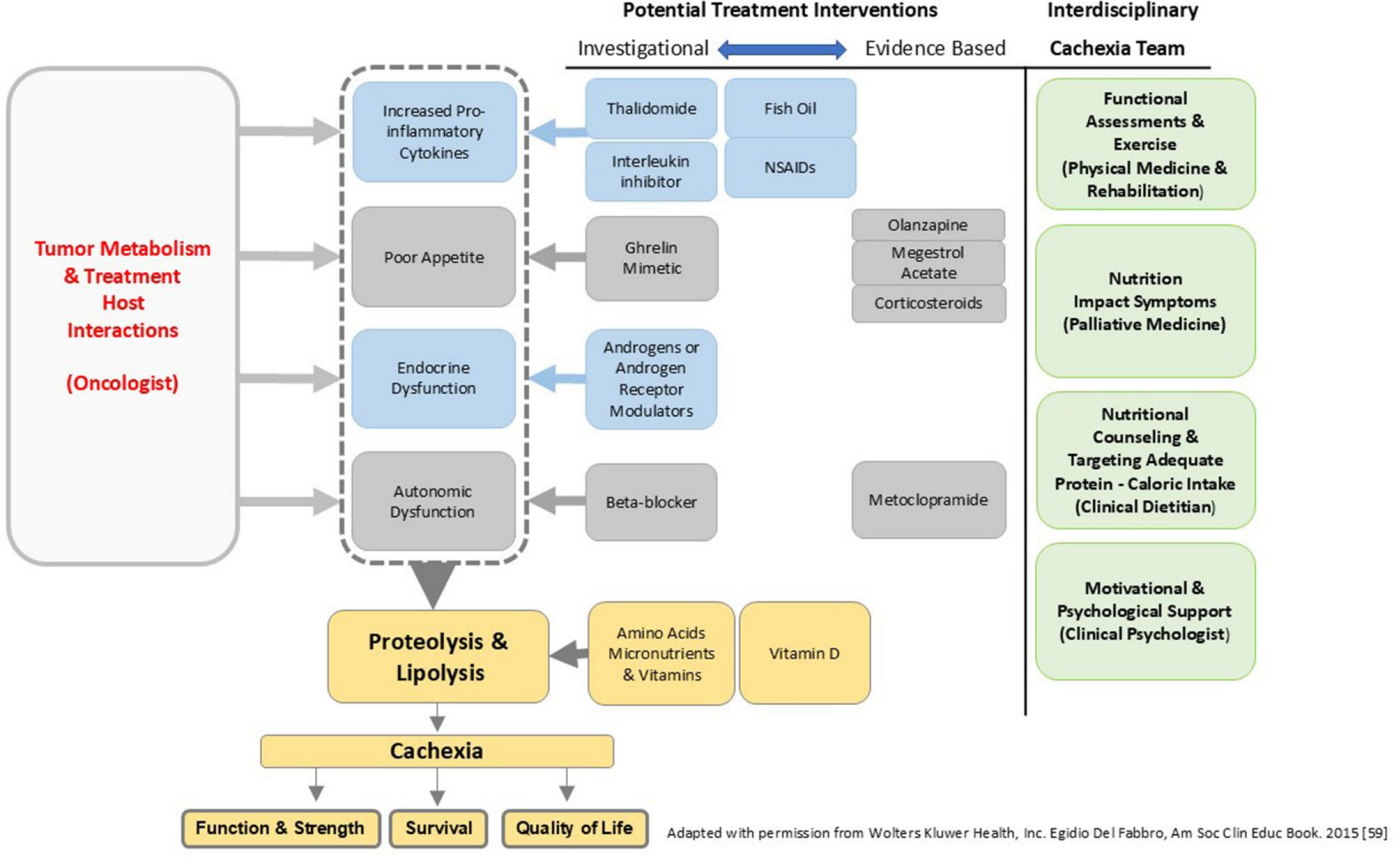
Multi-modality treatment for anorexia-cachexia syndrome in patients with cancer.

Trials combining exercise and nutrition plus an agent targeting one of the mechanisms of ACS, for example, NSAID for inflammation, have demonstrated feasibility and safety (Figure 1).^54^ Trials underway include a Korean RCT comparing multimodal intervention care (MIC) vs conventional palliative care. MIC includes ibuprofen, omega-3-fatty acid, oral nutritional supplement, weekly physical, psychiatric assessment, nutritional counseling, and complementary and alternative medicine.^55^ More trials evaluating anti-cachexia agents in combination with a multimodal approach are needed.^56^ Individualizing management of patients based on their symptom burden, function, pathophysiological drivers of cachexia, and cancer stage should be the aspirational model of care.

## Conclusion

ACS in patients with cancer is associated with increased physical and psychological symptom burden, loss of function, decreased QOL, and increased morbidity and mortality. To effectively treat ACS in patients with cancer, providers need to screen for malnutrition, evaluate body composition and functional status, and provide a thorough assessment and treatment of NIS. Given that multi-modality treatment is necessary to address all the clinical domains of ACS, the development of specialty clinics is desirable in cancer centers with adequate resources and expertise in treating ACS obtained from these pilot programs may be disseminated to other healthcare providers.

## References

[1] Roeland EJ , Bohlke K, Baracos VE, et al Management of cancer cachexia: ASCO guideline. J Clin Oncol. 2020;38:2438-2453. 10.1200/JCO.20.00611 32432946

[2] Nasrah R , Van Der Borch C, Kanbalian M, Jagoe RT. Defining barriers to implementation of nutritional advice in patients with cachexia. J Cachexia Sarcopenia Muscle. 2020;11:69-78. https://doi: 10.1002/jcsm.12490 31436033 10.1002/jcsm.12490 PMC7015253

[3] Dev R. Measuring cachexia-diagnostic criteria. Ann Palliat Med. 2019;8:24-32. 10.21037/apm.2018.08.07 30525765

[4] Blauwhoff-Buskermolen S , Ruijgrok C, Ostelo RW, et al The assessment of anorexia in patients with cancer: cut-off values for the FAACT-A/CS and the VAS for appetite. Support Care Cancer. 2016;24:661-666. https://doi: 10.1007/s00520-015-2826-2 26160463 10.1007/s00520-015-2826-2 PMC4689771

[5] Bauer J , Capra S, Ferguson M. Use of the scored patient-generated subjective global assessment (PG-SGA) as a nutrition assessment tool in patients with cancer. Eur J Clin Nutr. 2002;56:779-785. 10.1016/j.clnesp.2022.09.029 12122555

[6] Paur I , Smedshaug GB, Haugum B, et al The Norwegian Directorate of health recommends malnutrition screening tool (MST) for all adults. Clin Nutr ESPEN. 2022;52:28-31. https://doi: 10.1016/j.clnesp.2022.09.029 36513467 10.1016/j.clnesp.2022.09.029

[7] Boleo-Tome C , Chaves M, Monterio-Grillo I, et al Teaching nutrition integration: MUST screening in cancer. Oncologist. 2011;16:239-245. 10.1634/theoncologist.2010-0203 21273515 PMC3228088

[8] Molfino A , Imbimbo G, Laviano A. Current screening methods for the risk or presence of malnutrition in cancer patients. Cancer Manag Res. 2022;14:561-567. https://doi: 10.2147/CMAR.S294105 35210853 10.2147/CMAR.S294105 PMC8857947

[9] Martin L , Senesse P, Gioulbasanis I, et al Diagnostic criteria for the classification of cancer-associated weight loss. J Clin Oncol. 2015;33:90-99. 10.1200/JCO.2014.56.1894 25422490

[10] Yavuzsen T , Walsh D, Davis MP, et al Components of the anorexia-cachexia syndrome: gastrointestinal symptom correlates of cancer anorexia. Support Care Cancer. 2009;17:1531-1541. 10.1007/s00520-009-0623-5 19350287

[11] Quinten C , Coens C, Mauer M, et al EORTC Clinical Groups. Baseline quality of life as a prognostic indicator of survival: a meta-analysis of individual patient data from EORTC clinical trials. Lancet Oncol. 2009;10:865-871. https://doi: 10.1016/S1470-2045(09)70200-1. 19695956 10.1016/S1470-2045(09)70200-1

[12] Amano K , Morita T, Miura T, et al Impact of taste/smell disturbances on dietary intakes and cachexia-related quality of life in patients with advanced cancer. Support Care Cancer. 2023;31:141. 10.1007/s00520-023-07598-6 36715776

[13] Barajas Galindo DE , Vidal-Casariego A, Calleja-Fernández A, et al Appetite disorders in cancer patients: Impact on nutritional status and quality of life. Appetite. 2017;114:23-27. 10.1016/j.appet.2017.03.020 28315777

[14] Viana ECRM , Oliveira IDS, Rechinelli AB, et al Malnutrition and nutrition impact symptoms (NIS) in surgical patients with cancer. PLoS One. 2020;15:e0241305. 10.1371/journal.pone.0241305 33320857 PMC7737886

[15] Anandavadivelan P , Martin L, Djärv T, Johar A, Lagergren P. Nutrition impact symptoms are prognostic of quality of life and mortality after surgery for oesophageal cancer. Cancers (Basel). 2018;10:318. https://doi: 10.3390/cancers10090318 30205530 10.3390/cancers10090318 PMC6162430

[16] Farhangfar A , Makarewicz M, Ghosh S, et al Nutrition impact symptoms in a population cohort of head and neck cancer patients: multivariate regression analysis of symptoms on oral intake, weight loss and survival. Oral Oncol. 2014;50:877-883. https://doi: 10.1016/j.oraloncology.2014.06.009 25017804 10.1016/j.oraloncology.2014.06.009

[17] Nourissat A , Bairati I, Fortin A, et al Factors associated with weight loss during radiotherapy in patients with stage I or II head and neck cancer. Support Care Cancer. 2012;20:591-599. 10.1007/s00520-011-1132-x 21424341

[18] Martin L , Lagergren P. Risk factors for weight loss among patients surviving 5 years after esophageal cancer surgery. Ann Surg Oncol. 2015;22:610-616. 10.1245/s10434-014-3973-2 25120247

[19] Amano K , Morita T, Koshimoto S, et al Eating-related distress in advanced cancer patients with cachexia and family members: a survey in palliative and supportive care settings. Support Care Cancer. 2019;27:2869-2876. 10.1007/s00520-018-4590-6 30554279

[20] Amano K , Baracos VE, Morita T, et al The impact of cachexia on dietary intakes, symptoms, and quality of life in advanced cancer. JCSM Rapid Commun. 2022;5:162-170. 10.1002/rco2.61

[21] Khorasanchi A , Nemani S, Pandey S, Del Fabbro E. Managing nutrition impact symptoms in cancer cachexia: a case series and mini review. Front Nutr. 2022;9:831934. 10.3389/fnut.2022.831934 35308290 PMC8928189

[22] Andrew IM , Waterfield K, Hildreth AJ, Kirlpatrick G, Hawkins C. Quantifying the impact of standardized assessment and symptom management tools on symptoms associated with cancer-induced anorexia cachexia syndrome. Palliat Med. 2009;23:680-688. 10.1177/0269216309106980 19797339

[23] Del Fabbro E , Hui D, Dalal S, et al Clinical outcomes and contributors to weight loss in a cancer cachexia clinic. J Palliat Med. 2011;14:1004-1008. 10.1089/jpm.2011.0098 21793729 PMC3166181

[24] Herault A , Lévêque E, Draye-Carbonnier S, et al High prevalence of pre-existing sarcopenia in critically ill patients with hematologic malignancies admitted to the intensive care unit for sepsis or septic shock. Clin Nutr ESPEN. 2023;55:373-383. https://doi: 10.1016/j.clnesp.2023.04.007 37202070 10.1016/j.clnesp.2023.04.007

[25] Donini LM , Busetto L, Bauer JM, et al Critical appraisal of definitions and diagnostic criteria for sarcopenic obesity based on a systematic review. Clin Nutr. 2020;39:2368-2388. 10.1016/j.clnu.2019.11.024 31813698

[26] Aleixo GFP , Shachar SS, Nyrop KA, Muss HB, Battaglini CL, Williams GR. Bioelectrical impedance analysis for the assessment of sarcopenia in patients with cancer: a systematic review. Oncologist. 2020;25:170-182. https://doi: 10.1634/theoncologist.2019-0600 32043785 10.1634/theoncologist.2019-0600 PMC7011645

[27] McDonald J , Sayers J, Anker SD, et al Cancer Cachexia Endpoints Working Group Physical function endpoints in cancer cachexia clinical trials: Systematic review 1 of the cachexia endpoints series. J Cachexia Sarcopenia Muscle. 2023;14:1932-1948. Online ahead of print. 10.1002/jcsm.13321 37671529 PMC10570071

[28] Pandya C , Magnuson A, Flannery M, et al Association between symptom burden and physical function in older patients with cancer. J Am Geriatr Soc. 2019;67:998-1004. 30848838 10.1111/jgs.15864 PMC7851835

[29] Klepin HD , Geiger AM, Tooze JA, et al Geriatric assessment predicts survival for older adults receiving induction chemotherapy for acute myelogenous leukemia. Blood. 2013;121:4287-4294. 23550038 10.1182/blood-2012-12-471680 PMC3663423

[30] Cesari M , Cerullo F, Zamboni V, et al Functional status and mortality in older women with gynecological cancer. J Gerontol A Biol Sci Med Sci. 2013;68:1129-1133. VolumeIssueSeptember Pages 10.1093/gerona/glt073 23733856

[31] Collins JT , Noble S, Chester J, et al The value of physical performance measurements alongside assessment of sarcopenia in predicting receipt and completion of planned treatment in non-small cell lung cancer: an observational exploratory study. Support Care Cancer. 2018;26:119-127. 28721555 10.1007/s00520-017-3821-6

[32] Hopkinson JB. The emotional aspects of cancer anorexia. Curr Opin Support Palliat Care. 2010;4:254-258. 10.1097/SPC.0b013e32833ef813 20881500

[33] Strasser F , Binswanger J, Cerny T, Kesselring A. Fighting a losing battle: eating-related distress of men with advanced cancer and their female partners. A mixed-methods study. Palliat Med. 2007;21:129-137. 10.1177/0269216307076346 17344261

[34] Hopkinson JB. Food connections: a qualitative exploratory study of weight- and eating-related distress in families affected by advanced cancer. Eur J Oncol Nurs. 2016;20:87-96. 10.1016/j.ejon.2015.06.002 26088124

[35] Hopkinson JB. The psychosocial components of multimodal interventions offered to people with cancer cachexia: a scoping review. Asia Pac J Oncol Nurs. 2021;8:450-461. 10.4103/apjon.apjon-219 34527775 PMC8420917

[36] Del Fabbro E , Orr TA, Stella SM. Practical approaches to managing cancer patients with weight loss. Curr Opin Support Palliat Care. 2017;11:272-277. 10.1097/SPC.0000000000000300 28957881

[37] Baracos VE , Coats AJ, Anker SD, Sherman L, Klompenhouwer T; International Advisory Board, and Regional Advisory Boards for North America, Europe, and Japan. Identification and management of cancer cachexia in patients: assessment of healthcare providers’ knowledge and practice gaps. J Cachexia Sarcopenia Muscle. 2022;13:2683-2696. 10.1002/jcsm.13105 36218155 PMC9745486

[38] Amano K , Koshimoto S, Hopkinson JB, et al Perspectives of health care professional on multimodal interventions for cancer cachexia. Palliat Med Rep. 2022;3:244-254. 10.1089/pmr.2022.0045 36636614 PMC9811833

[39] Chasen MR , Bhargava R. A rehabilitation program for patients with gastroesophageal cancer—a pilot study. Support Care Cancer. 2010;18 Suppl 2:S35-40. 10.1007/s00520-010-0828-7 20177712

[40] Chasen MR , Feldstain A, Gravelle D, et al An interprofessional palliative care oncology rehabilitation program: effects on function and predictors of program completion. Curr Oncol. 2013;20:301-309. 10.3747/co.20.1607 24311945 PMC3851341

[41] Gagnon B , Murphy J, Eades M, et al A prospective evaluation of an interdisciplinary nutrition-rehabilitation program for patients with advanced cancer. Curr Oncol. 2013;20:310-318. 10.3747/co.20.1612 24311946 PMC3851342

[42] Eades M , Murphy J, Carney S, et al Effect of an interdisciplinary rehabilitation program on quality of life in patients with head and neck cancer: review of clinical experience. Head Neck. 2013;35:343-349. 10.1002/hed.22972 22422558

[43] Parmar MP , Vanderbyl BL, Kanbalian M, et al A multidisciplinary rehabilitation programme for cancer cachexia improves quality of life. BMJ Support Palliat Care. 2017;7:441-449. 10.1136/bmjspcare-2017-001382 28847854

[44] Vaughan VC , Farrell H, Lewandowski PA, McCoombe SG, Martin P. Defining a new model of interdisciplinary cancer cachexia care in regional Victoria, Australia. Support Care Cancer. 2020;28:3041-3049. 10.1007/s00520-019-05072-w 31578643

[45] Feldstain A , Lebel S, Chasen MR. An interdisciplinary palliative rehabilitation intervention bolstering general self-efficacy to attenuate symptoms of depression in patients living with advanced cancer. Support Care Cancer. 2016;24:109-117. 10.1007/s00520-015-2751-4 25953381

[46] Uster A , Ruehlin M, Mey S, et al Effects of nutrition and physical exercise intervention in palliative cancer patients: a randomized controlled trial. Clin Nutr. 2018;37:1202-1209. 10.1016/j.clnu.2017.05.027 28651827

[47] Lu Z , Fang Y, Liu C, et al Early interdisciplinary supportive care in patients with previously untreated metastatic esophagogastric cancer: a phase III randomized controlled trial. J Clin Oncol. 2021;39:748-756. 10.1200/JCO.20.01254 33417481 PMC8078238

[48] Barnes O , Wilson RL, Gonzalo-Encabo P, et al The effect of exercise and nutritional interventions on body composition in patients with advanced or metastatic cancer: a systematic review. Nutrients. 2022;14:2110. 10.3390/nu14102110 35631251 PMC9145470

[49] Carli F , Bousquet-Dion G, Awasthi R, et al Effect of multimodal prehabilitation vs postoperative rehabilitation on 30-day postoperative complications for frail patients undergoing resection of colorectal cancer: a randomized clinical trial. JAMA Surg. 2020;155:233-242. 10.1001/jamasurg.2019.5474 31968063 PMC6990653

[50] Phillips I , Petrie R, Allan L, et al Early prehabilitation in suspected locally advanced and metastatic lung cancer. BMJ Support Palliat Care. 2024;13:e908-e911. Jul 26: spcare-2023-004349. 10.1136/spcare-2023-004349 37495261

[51] Naito T , Mitsunaga S, Miura S, et al Feasibility of early multimodal interventions for elderly patients with advanced pancreatic and non‐small‐cell lung cancer. J Cachexia Sarcopenia Muscle. 2019;10:73-83. 30334618 10.1002/jcsm.12351 PMC6438328

[52] Hall CC , Cook J, Maddocks M, Skipworth RJE, Fallon M, Laird BJ. Combined exercise and nutritional rehabilitation in outpatients with incurable cancer: a systematic review. Support Care Cancer. 2019;27:2371-2384. 10.1007/s00520-019-04749-6 30944994 PMC6541700

[53] Senesse P , Isambert A, Janiszewski C, et al Management of cancer cachexia and guidelines implementation in a comprehensive cancer center: a physician-led cancer nutrition program adapted to the practices of a country. J Pain Symptom Manage. 2017;54:387-393.e3. 10.1016/j.jpainsymman.2017.01.010 28778558

[54] Solheim TS , Laird BJA, Balstad TR, et al Cancer cachexia: rationale for the MENAC (multimodal-exercise, nutrition and anti-inflammatory medication for cachexia) trial. BMJ Support Palliat Care. 2018;8:258-265. 10.1136/bmjspcare-2017-001440 29440149

[55] Maeng CH , Kim BH, Chon J, et al Effect of multimodal intervention care on cachexia in patients with advanced cancer compared to conventional management (MIRACLE): an open-label, parallel, randomized, phase 2 trial. Trials. 2022;23:281. 10.1186/s13063-022-06221-z 35410294 PMC8996396

[56] Mantovani G , Macciò A, Madeddu C, et al Randomized phase III clinical trial of five different arms of treatment in 332 patients with cancer cachexia. Oncologist. 2010;15:200-211. 10.1634/theoncologist.2009-0153. Epub 2010 Feb 15 20156909 PMC3227938

[57] Glare P , Jongs W, Zafiropoulos B. Establishing a cancer nutrition rehabilitation program (CNRP) for ambulatory patients attending an Australian cancer center. Support Care Cancer. 2011;19:445-454. 10.1007/s00520-010-0834-9 20204419

[58] Granda-Cameron C , DeMille D, Lynch MP, et al An interdisciplinary approach to manage cancer cachexia. Clin J Oncol Nurs. 2010;14:72-80. 10.1188/10.CJON.72-80 20118029

[59] Del Fabbro E. Current and future care of patients with the cancer anorexia-cachexia syndrome. Am Soc Clin Oncol Educ Book. 2015;e229-2237-e237. 10.14694/EdBook_AM.2015.35.e229 25993178

